# *A Posteriori* dietary patterns, insulin resistance, and diabetes risk by Hispanic/Latino heritage in the HCHS/SOL cohort

**DOI:** 10.1038/s41387-022-00221-3

**Published:** 2022-10-13

**Authors:** Luis E. Maldonado, Daniela Sotres-Alvarez, Josiemer Mattei, Martha L. Daviglus, Gregory A. Talavera, Krista M. Perreira, Linda Van Horn, Yasmin Mossavar-Rahmani, Madison N. LeCroy, Linda C. Gallo, Sandra S. Albrecht

**Affiliations:** 1 Carolina Population Center, University of North Carolina, Chapel Hill, NC USA; 2 Department of Nutrition, Gillings School of Global Public Health, University of North Carolina, Chapel Hill, NC USA; 3 Department of Biostatistics, Gillings School of Global Public Health, University of North Carolina, Chapel Hill, NC USA; 4 Department of Nutrition, Harvard T.H. Chan School of Public Health, Boston, MA USA; 5 Institute for Minority Health Research, University of Illinois College of Medicine, Chicago, IL USA; 6 Department of Psychology, San Diego State University, San Diego, CA USA; 7 Department of Social Medicine, University of North Carolina, Chapel Hill, NC USA; 8 Department of Preventive Medicine, Northwestern University, Chicago, IL USA; 9 Department of Epidemiology & Population Health, Albert Einstein College of Medicine, Bronx, NY USA; 10 Department of Epidemiology, Mailman School of Public Health, Columbia University, New York, NY USA

**Keywords:** Risk factors, Diabetes

## Abstract

**Objective:**

We examined links among dietary patterns (DPs), insulin resistance (IR), and diabetes risk by heritage in the Hispanic Community Health Study/Study of Latinos.

**Methods:**

Hispanics/Latinos of Cuban, Dominican, Mexican, Puerto Rican, Central American, and South American heritage aged 18–74 years and diabetes-free completed two 24 h dietary recalls at baseline (2008–2011) and provided 6-year follow-up data (2014–2017; *n* = 7774). We classified 6-year IR status [improved, unchanged (referent), worsened] using a 1-SD change in fasting insulin between visits and defined incident diabetes based on American Diabetes Association criteria. We derived heritage-specific DPs via principal factor analysis and estimated their associations with 6-year IR status (multinomial) and incident diabetes (binary) using complex survey-based logistic regression.

**Results:**

Five overarching DPs based on high-loading foods were shared by two or more heritage groups: “Burger, Fries, & Soft Drinks”; “White Rice, Beans, & Red Meats”; “Fish & Whole Grains”; “Cheese & Sweets”; and “Stew & Corn”. Comparing highest-to-lowest DP quintiles, the Dominican “Burger, Fries, & Soft Drinks” and Cuban “White Rice, Beans, & Red Meats” DPs were associated with worsened 6-year IR status (log-odds: 2.35, 95% CI: 1.02, 3.68, *P*_trend_ = 0.037 and log-odds: 1.27, 95% CI: 0.49, 2.06, *P*_trend_ = 0.009, respectively). The Puerto Rican “Burger, Fries, & Soft Drinks” and the Central American “White Rice, Beans, & Red Meats” DPs were associated with greater diabetes incidence (OR: 3.00, 95% CI:1.50, 5.99 and OR: 2.41, 95% CI: 1.05, 5.50, respectively).

**Conclusions:**

A diet characterized by higher intakes of burgers, fries, and soft drinks and another characterized by higher intakes of white rice, beans, and red meats may be adversely associated with IR and diabetes risk in some Hispanic/Latino heritage groups. Future work is needed to offer more heritage-specific dietary guidance for diabetes prevention in this population.

## Introduction

In the US, diabetes disproportionately affects Hispanics/Latinos [1]. Disparities by heritage among Hispanics/Latinos have also been reported with prevalence estimates highest in individuals of Mexican heritage (14.4%) and lowest in those of Cuban heritage (6.5%) [2, 3]. However, the reasons for this disparity are unknown. Diet, a strong predictor of diabetes, has been shown to vary by heritage [4] and may partly explain these disparities in diabetes prevalence among US Hispanics/Latinos [3].

Despite social, cultural, and genetic diversity among Hispanics/Latinos [4–6], few studies have evaluated links between diet and diabetes and intermediary pathways such as insulin resistance (IR) [7] among different groups of Hispanics/Latinos. Previous research in this population has been cross-sectional, and defined diets using a priori indexes [6, 8] or individual foods/nutrients [9], neither of which necessarily capture commonly consumed foods and culturally-relevant dietary behaviors (e.g., types of foods consumed together) specific to different groups of Hispanics/Latinos. Previous research using data from the Hispanic Community Health Study/Study of Latinos (HCHS/SOL) found substantial variation in the food group composition of *a posteriori* heritage-specific dietary patterns (DPs) in six large heritage groups (Cuban, Dominican, Mexican, Puerto Rican, Central American, and South American) [10]. Whether this variation in DPs by heritage differentially predicts diabetes risk and IR status warrants investigation.

Most *a posteriori* DPs previously linked to diabetes risk (e.g. Unhealthy/Western and Healthy/Prudent) [11] were derived using predominantly non-Hispanic white samples, limiting their generalizability. There may be, however, other *a posteriori* DPs relevant to diabetes, particularly among high-risk populations such as Hispanics/Latinos, that remain unidentified [12]. Additionally, some *a posteriori* DPs (e.g., Western) have been shown to contribute to IR with associations not fully explained by obesity, measured using body mass index (BMI) [7]. Whether waist circumference, a relatively stronger predictor of diabetes versus BMI, better explains associations between DPs and IR merits further investigation. Findings could potentially inform preventive interventions targeting diabetes in this diverse US population.

Using data from the Hispanic Community Health Study/Study of Latinos (HCHS/SOL), we first characterized heritage-specific *a posteriori* DPs derived separately in six heritage groups among individuals without diabetes at baseline. Although heritage-specific DPs had been previously derived in HCHS/SOL, individuals with diabetes were included in those analyses [10] and, subsequently, may have influenced resulting DPs, since people with diabetes tend to adopt healthier diets in response to doctors’ recommendations [9]. We then examined associations between heritage-specific DPs at baseline and 6-year IR status and diabetes incidence within each heritage group; and whether waist circumference contributed to these associations.

## Methods

### Study population

The HCHS/SOL is a population-based cohort study of 16,415 Hispanic/Latinos aged 18–74 years living in 4 US cities (Bronx, NY; Chicago, IL; Miami, FL; and San Diego, CA). Participants were recruited by using a 2-stage probability sample design, as described elsewhere [13, 14]. The baseline examination (2008–2011) included comprehensive biological, behavioral, and socio-demographic assessments. Roughly 6 years later, 11,623 participants (70.8%) returned for a second in-clinic visit (2014–2017). Annual follow-up telephone calls ascertained information on cardiopulmonary outcomes and diabetes onset. The study was approved by the Institutional Review Boards (IRBs) at all participating institutions, and all participants gave written informed consent.

### Insulin resistance and diabetes status definition

During both visits, blood samples were collected by a venous puncture after a fasting period ≥8 h prior to the visit. Data on fasting glucose, fasting insulin, and hemoglobin A1C (HbA1C) were collected. Study procedures included a 2 h oral glucose tolerance test (OGTT). For safety reasons, the protocol specified not administering the OGTT among individuals with fasting plasma glucose (FPG) ≥ 150 mg/dL, previous diabetes diagnosis, or medications for diabetes treatment. The assays’ methodologies/procedures are described on the HCHS/SOL website (https://sites.cscc.unc.edu/hchs/node/4055). Participants were defined as having diabetes based on self-reported physician-diagnosed diabetes, documented use of diabetes medications [15], and lab data meeting any of the following criteria: fasting plasma glucose (≥126 mg/dL), post-OGTT (≥200 mg/dL), or HbA1C (≥6.5%). For all analyses, we excluded participants with diabetes at baseline. To assess incident diabetes, we applied these same criteria to determine diabetes status at visit 2, supplemented with self-report of diabetes onset from the annual follow-up telephone calls. We used fasting insulin to measure IR. Compared to other surrogate measures (e.g., HOMA-IR), change in fasting insulin is more interpretable, and has been widely used to evaluate IR in population-based studies [16]. Change in insulin was first calculated using the difference between visit values (visit 2 − visit 1). We then categorized these differences using a change in standard deviation (SD) to reflect improved (≤ −1 SD), unchanged (−1 > SD < 1), or worsened (≥1 SD) IR status throughout the 6-year follow-up.

### Dietary assessment

Methods for dietary data collection have been published [4]. Briefly, two, non-consecutive 24 h dietary recalls were administered by centrally trained bilingual registered dietitians, the first in person at the baseline visit, and the second via telephone ~5–90 days later. Dietary assessment was conducted using the multiple-pass methods described by the Nutrition Data System for Research (NDS-R) software (version 11) from the Nutrition Coordinating Center, University of Minnesota. NDSR includes Hispanic and Latino foods.

We formed 34 food groups according to previously documented methodology [10]. Briefly, food groups were based on cultural and behavioral relevance, previous work (e.g., corn-based foods, burgers, meat and vegetable stews) [10, 17, 18], and consumption patterns in the data (Supplemental Table S1). For instance, we grouped ingredients (e.g., corn tortilla, beef, onion) of mixed dishes (e.g., taco) together to reflect real eating behaviors in which foods are consumed together. Whole/non-recipe foods (e.g., chocolate) were classified based on the University of North Carolina food grouping system, which disaggregates the major US Department of Agriculture’s food groups by fat and fiber [19]. Due to low consumption of some foods in some heritage groups, we aggregated low- and high-nutrient specific food groups (e.g., high- and low-fat milk into milk). Additionally, we separated fried from non-fried foods (e.g., fried vs. grilled chicken) across food groups except for corn-based foods, which traditionally include fried/grilled corn tortillas. Food group intakes (grams/day) [20, 21] were summed in each 24 h recall and then averaged for each participant. To address skewness due to high proportions of non-consumers of episodically consumed foods, we classified food group intakes into 3-level ordinal variables (non-consumers, and below and above the median). Food groups with consumption <5% in at least one heritage group were either merged with another food group or dropped from analysis.

### Alternative healthy eating index (AHEI-2010)

To assess “healthfulness” of each *a posteriori* heritage-specific DPs, we used the AHEI-2010, a diet quality index previously linked to cardiometabolic disease risk [22]. Previously established approaches were used to construct the AHEI-2010 score, which range from 0 (lowest diet quality) to 110 (highest diet quality) [6].

### Hispanic/Latino heritage and covariates

Questionnaires were interviewer-administered at both visits in English or Spanish. Hispanic/Latino heritage was self-reported at baseline from a list of heritage groups, including Cuban, Dominican, Mexican, Puerto Rican, Central American, South American, more than one heritage, and other. Other sociodemographic data collected included age at examination (years), sex (male, female), highest education achieved (less than high school (HS) diploma, HS diploma or equivalent, beyond HS), and nativity (US-born (only includes 50 states and DC)/foreign-born)) [23]. Physical activity was collected at baseline using a modified Global Physical Activity Questionnaire, which is designed to assess average time and number of days spent on a given activity in work, travel, and leisure domains [24, 25]. Data were summarized in metabolic equivalent-minutes/day and categorized as low and moderate/vigorous [24]. Smoking status at baseline was also collected using interviewer-administered questionnaires (never, former, current, missing). Waist circumference (WC) was measured using anthropometric tape at the horizontal line just above the uppermost lateral border of the right ilium. We calculated total elapsed time (years) between visits by taking the difference between visit 1 and visit 2 dates and dividing by 365.25.

### Dietary pattern analysis

To empirically derive *a posteriori* heritage-specific factors, or DPs, we used principal factor analysis (PFA) among individuals without baseline diabetes (Supplemental Fig. S1). Because PFA requires a large sample size for stable correlations among input variables [26], we included all subjects that met the minimum inclusion criteria (e.g. no baseline diabetes, Cuban, Dominican, Mexican, Puerto Rican, Central American, or South American heritage), and two energy-plausible 24 h recalls). This resulted in a PFA sample of 11,125 which was larger than the main analyses of IR and diabetes risk (details in next section). First, we generated matrices of polychoric correlations among the 34 food groups in each heritage group, and then performed PFA on each heritage-specific matrix with orthogonal (varimax) rotation to improve interpretability and minimize correlations of derived factors [27]. Factors emerged in decreasing order of the amount of variance explained, and factor retention was guided using a modified Delphi approach, a widely used method soliciting the opinions of experts to establish a convergence of opinion [28]. Three investigators met to reconcile differences in factor retention in each heritage group based on scree plots (Supplemental Fig. S2), factor loadings, variance explained, interpretability of factors, and consistency with the literature [29]. We generated DP scores by multiplying the scoring coefficient of each food group by the individual’s corresponding food group intake category (non-consumer, below or above the median) and summing across food groups. Consistent with previous work [10], we identified overarching DPs based on 2–3 food groups with similar high factor loadings (>0.20) [11, 30] shared by two or more heritage groups. Although we understand 2–3 dietary components do not comprehensively characterize a dietary pattern, to be clear and succinct about prominent food combinations reflected in our dietary findings, we opted for this labeling approach. Lastly, we categorized heritage-specific DP scores using unweighted quintiles and evaluated mean AHEI-2010 scores by quintiles to further characterize DPs.

Among the PFA-eligible sample, we derived 19 heritage-specific DPs (4 in Cuban; 2 in Dominican; 3 in Mexican; 4 in Puerto Rican; 3 in Central American; 3 in South American) that contributed to 5 overarching DPs based on high-loading foods shared by two or more heritage groups (Supplemental Table S3). Burgers, fries, soft drinks, and pizza had high loadings for the first factor derived in each heritage group, except among South Americans, whose second factor loaded highly on these foods. We therefore classified this factor as the “Burger, Fries, & Soft Drinks” DP, which included the same foods across heritage groups. The other DPs were more heritage-specific, as the number of foods shared by different heritage groups decreased “White Rice, Beans, & Red Meats” DP was characterized by high loadings on white rice, beans, and red meats (pork or beef) in all except the South American group. The “Fish & Whole Grain” DP was characterized by fish and whole grains in Cuban, Mexican, Puerto Rican, and South American groups. The “Cheese & Sweets” DP included cheese, sweets, noodle-based foods, and fried foods in Cuban and South American groups; and a “Stew & Corn” DP included stews and corn-based foods in Puerto Rican and Central American groups. The proportion of the variance explained by heritage-specific factors ranged from 8.1 to 26.6%.

### Statistical analysis

**Fig. 1:**
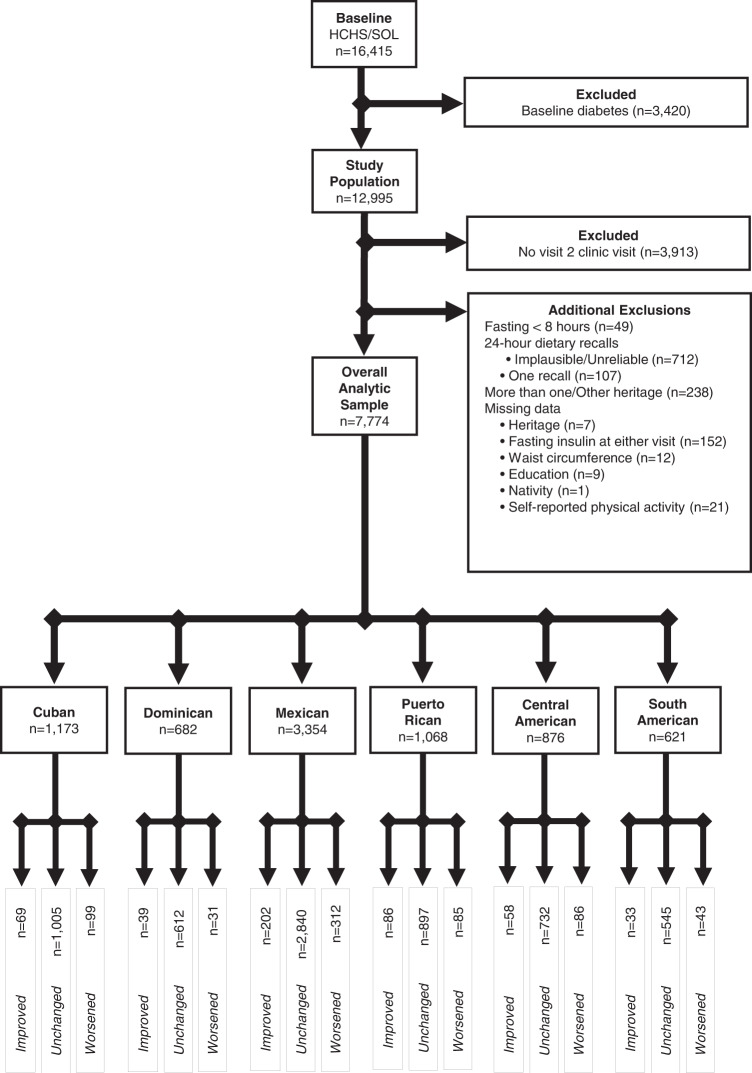
Flowchart of heritage-specific analytic samples by insulin resistance status at 6 years in HCHS/SOL^a^. ^a^Hispanic Community Health Survey/Study of Latinos, HCHS/SOL.

All analyses accounted for the complex survey design and sampling weights using Stata version 14.2 (Stata Corp, College Station, Texas). Of the 16,415 adults at baseline, we excluded participants with diabetes at baseline (*n* = 3420), no follow-up visit (*n* = 3913), fasted <8 h prior to either visit (*n* = 49), did not have two 24 h dietary recalls with plausible energy values (kcals > 500 or kcals<5000; *n* = 819), self-reported more than one or “other” heritage (*n* = 238) and had missing data on heritage (*n* = 7), insulin (*n* = 152) and other covariates (*n* = 43), yielding a final sample size of 7774 (Fig. 1, flowchart).

We tested differences in sociodemographic and health characteristics by heritage using analysis of variance for continuous variables and Pearson chi-square for categorical variables. We then tested mean differences in AHEI-2010 score by quintiles of each DP to assess correlations with a measure of diet “healthfulness” using linear regression, adjusting for age, sex, and education. To prospectively evaluate associations between baseline DPs and IR status, we used multivariable multinomial logistic regression to estimate 6-year log-odds (likelihood) of worsened and improved (versus unchanged) insulin levels comparing highest-to-lowest quintiles of DPs in each heritage group. We used multivariable logistic regression to assess relationships between DPs and incident diabetes (cumulative incident proportion). Model 1 adjusted for elapsed time between visits and baseline covariates, including age, sex, education, nativity, total energy intake, natural log of fasting insulin (except incident diabetes models), and physical activity. Estimates did not change when we also adjusted for smoking status, thus, it was not included in final models (data not shown). To better understand the direct contributions of diet on the outcomes independent of central adiposity, Model 2 was further adjusted for WC. Because participants with incident diabetes may have been taking antidiabetic medications at visit 2, impacting insulin levels, we performed a sensitivity analysis excluding participants taking antidiabetic medication at either visit (*n* = 10). Finally, to assess potential for selection bias, we conducted sensitivity analyses by testing baseline sociodemographic and AHEI-2010 score differences between our analytic sample and participants without baseline diabetes who were excluded from our analysis. *P*_trend_ values were calculated by including the midpoint of each quintile of each dietary pattern as a continuous variable (score units). We set statistical significance at *p* < 0.05.

## Results

### Sample characteristics

Table 1 shows baseline characteristics and 6-year diabetes incidence by heritage among the main analysis sample (*n* = 7774). Individuals of Cuban heritage were the oldest on average, and individuals of Mexican heritage were the youngest. A larger proportion of all heritage groups, except Puerto Rican, were foreign-born. Cuban and South American groups had the highest proportions of individuals with more than high school education. The Cuban group also had the lowest proportion of individuals engaging in moderate/vigorous physical activity and the highest mean total energy compared to others. The Puerto Rican group had the highest mean WC and lowest mean AHEI-2010 scores. While the Dominican and South American groups had the lowest geometric mean fasting insulin at baseline, the Cuban and Central American groups had the highest. The 6-year incident proportion of diabetes was highest among the Puerto Rican group and lowest in the Central American group. Compared to the analytic sample, excluded individuals were significantly younger, male, US-born, had an education beyond high school, and worse diet quality scores (Supplemental Table S2).

**Table 1:** Baseline characteristics and 6-year diabetes incidence by Hispanic/Latino heritage group in HCHS/SOL (*n* = 7774)^a^.

Baseline characteristics	Cuban	Dominican	Mexican	Puerto Rican	Central American	South American	*P*
	*n* = 1173	*n* = 682	*n* = 3354	*n* = 1068	*n* = 876	*n* = 621	
*Sociodemographic*							
Age (years)	44.6 ± 0.7	37.8 ± 0.8	36.6 ± 0.4	41.0 ± 0.7	37.4 ± 0.6	41.1 ± 0.9	<0.001
Female (%)	48.0	57.4	53.5	48.1	50.5	54.6	0.007
Nativity (%)							
US-born	8.3	13.3	24.1	54.3	6.6	5.5	<0.001
Foreign-born	91.7	86.7	75.9	45.7	93.4	94.5	
Education (%)							
<High school	17.1	36.4	30.3	33.1	39.4	18.3	<0.001
High school/equivalent	29.0	22.3	33.7	27.8	24.2	26.1	
>High school	54.0	41.3	36.0	39.1	36.4	55.6	
Time between visits, years	5.9 ± 0	6.1 ± 0	6.2 ± 0	6.2 ± 0	6.0 ± 0.1	5.9 ± 0.1	<0.001
*Health characteristic*							
MV physical activity (%)^a,b^	50.3	61.3	64.5	62.3	59.5	59.6	<0.001
Smoking status							<0.001
Never	58.7	81.3	67.7	52.8	72.1	69.4	
Former	16.5	9	16.6	15.7	13.4	20.9	
Current	24.7	9.7	15.7	31.5	14.4	9.6	
Missing	0	0	0.1	0	0.1	0.2	
Waist circumference (cm)	96 ± 0.6	94 ± 0.8	96.8 ± 0.4	97.6 ± 0.7	93.1 ± 0.5	92.7 ± 0.6	<0.001
Total energy (kJ/day)	9015 ± 143	6964 ± 177	8311 ± 87	7955 ± 137	8019 ± 175	7947 ± 160	<0.001
AHEI-2010 (score units)^a^	43.6 ± 0.2	48.2 ± 0.3	51.6 ± 0.2	41.4 ± 0.3	46.7 ± 0.3	45.7 ± 0.3	<0.001
Ln (fasting insulin (pmol/L))^c^	4.29 ± 0.03	4.09 ± 0.04	4.21 ± 0.03	4.21 ± 0.03	4.3 ± 0.03	4.16 ± 0.03	<0.001
Fasting glucose (mg/dL)	94.8 ± 0.3	91.3 ± 0.5	93 ± 0.2	93.5 ± 0.4	93.5 ± 0.4	93.5 ± 0.4	<0.001
Incident diabetes at 6 years	12.1	9.3	14.0	17.0	6.9	8.1	<0.001

Values are means ± SEs unless otherwise specified. All analyses were survey-weighted. Sample sizes are unweighted.

^a^*HCHS/SOL* Hispanic Community Health Survey/Study of Latinos, *MV* Moderate/vigorous, *AHEI* Alternative Healthy Eating Index.

^b^MV physical activity was self-reported at baseline and based on days and total metabolic equivalents (minutes/day) assessed using a modified Global Physical Activity Questionnaire, which asks about activity and inactivity (i.e. sedentary behavior) in several life domains (work, travel, leisure).

^c^Natural log (Ln) of baseline fasting insulin (pmol/L).

### Baseline overarching dietary patterns and AHEI-2010

Of the 5 overarching DPs, higher quintiles of the “Burger, Fries, & Soft Drinks” DPs were associated with lower mean scores on the AHEI-2010 in all heritage groups, except South American (Supplemental Fig. S3). In contrast, higher quintiles of the “Fish & Whole Grain” DPs in all heritage groups were associated with higher mean AHEI-2010 scores. For the other three DPs, the direction of the associations with AHEI-2010 depended on the heritage group. For instance, positive linear trends were observed for the Mexican “White Rice, Beans, & Red Meats” DP with AHEI-2010, but no associations were found for other heritage groups. The highest quintiles for the Cuban “Cheese & Sweets” and Puerto Rican “Stew & Corn” DPs were also associated with higher mean AHEI-2010 scores, but similar patterning did not extend to other heritage groups.

### Insulin resistance status by baseline dietary patterns

**Fig. 2:**
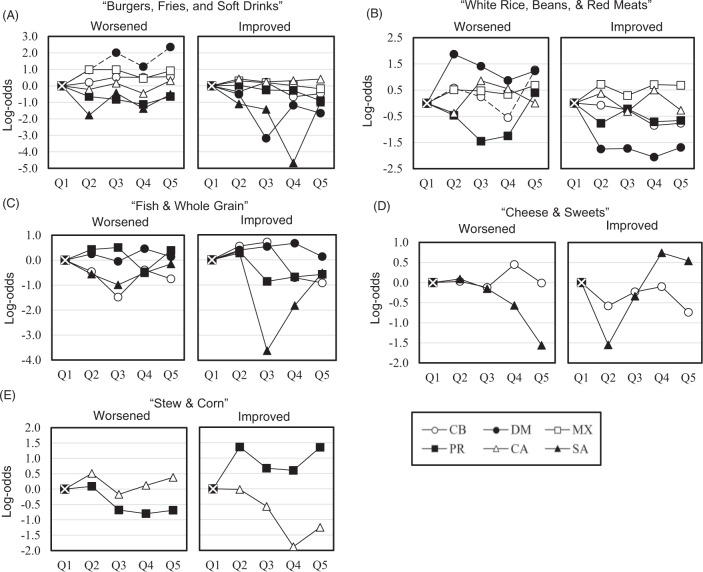
Six-year likelihood of worsened or improved (versus unchanged) insulin resistance status comparing highest-to-lowest quintiles of heritage-specific *a posteriori* dietary patterns for each overarching dietary pattern identified in HCHS/SOL^a^. ªLikelihood estimates are log-odds of worsened and improved (versus unchanged) insulin resistance status by heritage-specific *a posteriori* dietary patterns for each overarching dietary pattern identified in HCHS/SOL among the following heritage groups: Cuban (CB, *n* = 1173), Dominican (DM, *n* = 682), Mexican (MX, *n* = 3354), Puerto Rican (PR, *n* = 1068), Central American (CA, *n* = 876), South American (SA, *n* = 621). All models were survey weighted. Change in insulin levels was first calculated using the difference between visit values (visit 2 − visit 1) and then categorized (after showing no evidence of skewness) using a change in standard deviation (SD) to reflect (≤−1 SD), unchanged (−1 > SD < 1), or worsened (≥1 SD) insulin resistance status at 6 years. Models adjust for elapsed time between visits (years) and relevant baseline covariates, including age (years), sex (male, female), self-reported highest education achieved (<high school, high school or equivalent, >high school), nativity (US-born (US states only), foreign-born), total energy intake (kJ/day, natural log of baseline fasting insulin (pmol/L), and baseline self-reported physical activity level (low, moderate/vigorous) based on days and total metabolic equivalents (minutes/day) assessed using a modified Global Physical Activity Questionnaire, which asks about activity and inactivity (i.e. sedentary behavior) in several life domains (work, travel, leisure). *P*_trend_ values were calculated by including the midpoint of each quintile of each heritage-specific *a posteriori* dietary pattern as a continuous variable (score units). Dashed lines indicate significant linear trends and differences between highest-to-lowest quintiles at *P* < 0.05.

Figure 2 shows adjusted likelihood estimates (log-odds) of having worsened or improved (versus unchanged) IR status 6 years later, comparing highest-to-lowest quintiles of *a posteriori* heritage-specific DPs at baseline. Most DPs were not associated with IR status with some exceptions. The highest-versus-lowest quintiles of the Dominican “Burger, Fries, & Soft Drinks” DP and the Cuban “White Rice, Beans, & Red Meats” DPs were associated with greater likelihood of having worsened (versus unchanged) IR status (Dominican log-odds: 2.35, 95% CI: 1.02, 3.68, *P*_trend_ = 0.037; and Cuban log-odds: 1.27, 95% CI: 0.49, 2.06, *P*_trend_ = 0.009). This same DP in the Cuban group was also associated with lower likelihood of improved (versus unchanged) IR status (log-odds: −1.69, 95% CI: −3.25, −0.13*, P*_trend_ = 0.035). Findings were null for the other DPs. Estimates were unchanged after adjusting for WC across heritage-specific models (data not shown). Findings were also similar after excluding participants who reported taking diabetes medication (data not shown).

### Incident diabetes by baseline dietary patterns

Table 2 shows adjusted heritage-specific odds ratios of 6-year diabetes incidence for the five overarching DPs. Comparing highest-to-lowest quintiles, the “Burger, Fries, & Soft Drinks” DP only in the Puerto Rican group and the “White Rice, Beans, & Red Meats” DP only in the Central American group were associated with greater odds of developing diabetes 6 years later (OR: 3.00, 95% CI: 1.50, 5.99 and OR: 2.41, 95% CI: 1.05, 5.50, respectively). After adjusting for WC (Model 2), only findings for the “Burger, Fries, & Soft Drinks” DP in the Puerto Rican group remained statistically significant (OR: 2.63, 95% CI: 1.29, 5.36). Although none of the “Fish & Whole Grain” DPs were statistically significantly associated with incident diabetes, higher quintiles were generally associated with decreased 6-year diabetes incidence except for the Cuban heritage group for whom findings were in the opposite direction. Lastly, associations for the “Cheese & Sweets” and “Stew & Corn” DPs were generally null, except for the Cuban “Cheese & Sweets” DP, which was found significantly associated with reduced diabetes risk only after adjusting for WC (Model 2, OR: 0.43, 95% CI: 0.20, 0.94). Model estimates did not change when we adjusted for fasting glucose at baseline (data not shown).

**Table 2:** Odds ratios (ORs) of 6-year incident diabetes by quintiles of heritage-specific *a posteriori* dietary patterns for each overarching dietary pattern identified in HCHS/SOL^a^.

Overarching dietary patterns	Q1	Q2	Q3	Q4	Q5
	Ref	OR (95% CI)	OR (95% CI)	OR (95% CI)	OR (95% CI)
“Burger, Fries, & Soft Drinks”					
Cuban					
Model 1^b^	1.00	0.75 (0.42,1.34)	0.69 (0.36, 1.31)	0.96 (0.53,1.74)	0.70 (0.36,1.37)
Model 2^c^	1.00	0.80 (0.43, 1.49)	0.71 (0.37, 1.38)	1.00 (0.55, 1.82)	0.67 (0.33, 1.36)
Dominican					
Model 1	1.00	1.35 (0.56, 3.27)	1.48 (0.60, 3.63)	1.42 (0.59, 3.41)	1.71 (0.68, 4.33)
Model 2	1.00	1.36 (0.57, 3.28)	1.48 (0.61, 3.58)	1.33 (0.55, 3.24)	1.53 (0.59, 3.96)
Mexican^*^					
Model 1	1.00	0.88 (0.57, 1.35)	1.30 (0.82, 2.08)	1.31 (0.85, 2.03)	1.10 (0.68, 1.76)
Model 2	1.00	0.82 (0.53, 1.28)	1.20 (0.74, 1.97)	1.35 (0.86, 2.11)	1.05 (0.65, 1.69)
Puerto Rican					
Model 1	1.00	1.63 (0.90, 2.95)	1.22 (0.67, 2.22)	2.36 (1.18, 4.71)	3.00 (1.50, 5.99)
Model 2	1.00	1.50 (0.83, 2.70)	1.19 (0.64, 2.22)	2.30 (1.13, 4.72)	2.63 (1.29, 5.36)
Central Am.					
Model 1	1.00	1.31 (0.61, 2.81)	0.83 (0.32, 2.14)	1.41 (0.55, 3.63)	2.30 (0.77, 6.83)
Model 2	1.00	1.17 (0.53, 2.59)	0.75 (0.30, 1.90)	1.03 (0.36, 2.90)	1.98 (0.66, 5.95)
South Am.					
Model 1	1.00	1.47 (0.56, 3.83)	0.95 (0.40, 2.24)	0.52 (0.17, 1.55)	0.69 (0.24, 2.02)
Model 2	1.00	1.54 (0.58, 4.09)	0.95 (0.39, 2.29)	0.52 (0.16, 1.73)	0.72 (0.23, 2.23)
“White Rice, Beans, & Red Meats”					
Cuban					
Model 1	1.00	1.14 (0.61, 2.12)	1.13 (0.50, 2.57)	1.54 (0.79, 2.98)	1.66 (0.85, 3.25)
Model 2	1.00	1.09 (0.57, 2.08)	1.13 (0.50, 2.52)	1.43 (0.73, 2.81)	1.58 (0.80, 3.12)
Dominican					
Model 1	1.00	1.45 (0.58, 3.66)	1.58 (0.53, 4.71)	1.26 (0.43, 3.67)	1.72 (0.65, 4.55)
Model 2	1.00	1.76 (0.70, 4.46)	1.78 (0.59, 5.36)	1.50 (0.52, 4.31)	2.16 (0.83, 5.67)
Mexican					
Model 1	1.00	1.14 (0.70, 1.85)	1.31 (0.77, 2.23)	1.21 (0.78, 1.88)	1.18 (0.71, 1.96)
Model 2	1.00	1.14 (0.70, 1.86)	1.31 (0.77, 2.24)	1.17 (0.74, 1.83)	1.10 (0.65, 1.88)
Puerto Rican					
Model 1	1.00	0.38 (0.19, 0.77)	1.22 (0.64, 2.31)	0.73 (0.36, 1.48)	0.64 (0.31, 1.31)
Model 2	1.00	0.37 (0.18, 0.78)	1.20 (0.62, 2.30)	0.75 (0.37, 1.52)	0.65 (0.30, 1.39)
Central Am.					
Model 1	1.00	1.92 (0.81, 4.51)	1.96 (0.78, 4.93)	2.93 (1.11, 7.74)	2.41 (1.05, 5.50)*
Model 2	1.00	2.05 (0.81, 5.19)	1.72 (0.69, 4.28)	2.58 (0.96, 6.91)	2.12 (0.89, 5.02)
“Fish & Whole Grain”					
Cuban					
Model 1	1.00	1.01 (0.47, 2.16)	2.60 (1.23, 5.51)	1.48 (0.74, 2.94)	1.50 (0.60, 3.73)
Model 2	1.00	0.96 (0.44, 2.10)	2.63 (1.25, 5.58)	1.43 (0.71, 2.87)	1.37 (0.55, 3.45)
Mexican					
Model 1	1.00	0.82 (0.53, 1.28)	0.85 (0.55, 1.31)	1.28 (0.84, 1.95)	0.83 (0.52, 1.30)
Model 2	1.00	0.84 (0.54, 1.32)	0.89 (0.57, 1.38)	1.28 (0.83, 1.99)	0.89 (0.56, 1.42)
Puerto Rican					
Model 1	1.00	0.74 (0.36, 1.52)	0.58 (0.31, 1.07)	0.67 (0.36, 1.27)	0.67 (0.33, 1.37)
Model 2	1.00	0.74 (0.34, 1.61)	0.54 (0.29, 1.02)	0.63 (0.33, 1.21)	0.62 (0.30, 1.28)
South Am.					
Model 1	1.00	0.42 (0.15, 1.19)	0.85 (0.30, 2.42)	0.61 (0.20, 1.83)	0.50 (0.18, 1.42)
Model 2	1.00	0.54 (0.19, 1.52)	1.06 (0.39, 2.90)	0.83 (0.28, 2.45)	0.76 (0.28, 2.06)
“Cheese & Sweets”					
Cuban					
Model 1	1.00	0.75 (0.35, 1.60)	0.72 (0.34, 1.50)	0.87 (0.42, 1.83)	0.48 (0.23, 1.00)
Model 2	1.00	0.80 (0.38, 1.69)	0.69 (0.32, 1.47)	0.78 (0.36, 1.70)	0.43 (0.20, 0.94)*
South Am.					
Model 1	1.00	1.24 (0.39, 3.97)	1.37 (0.42, 4.52)	1.68 (0.60, 4.71)	1.07 (0.33, 3.52)
Model 2	1.00	1.32 (0.40, 4.34)	1.59 (0.49, 5.17)	2.01 (0.72, 5.65)	1.19 (0.36, 3.99)
“Stew & Corn”					
Puerto Rican					
Model 1	1.00	1.70 (0.84, 3.44)	0.79 (0.39, 1.60)	0.71 (0.35, 1.43)	0.98 (0.51, 1.87)
Model 2	1.00	1.56 (0.72, 3.40)	0.71 (0.34, 1.50)	0.63 (0.29, 1.33)	0.94 (0.47, 1.86)
Central Am.					
Model 1	1.00	1.31 (0.47, 3.61)	1.39 (0.61, 3.19)	1.32 (0.58, 2.97)	1.02 (0.41, 2.57)
Model 2	1.00	1.33 (0.46, 3.78)	1.42 (0.63, 3.20)	1.45 (0.61, 3.42)	1.07 (0.38, 2.95)

Data are odds ratios (ORs, 95% CI) of 6-year incident diabetes comparing highest-to-lowest (Q5 vs. Q1) quintiles of each heritage-specific *a posteriori* dietary pattern for each overarching dietary pattern identified in HCHS/SOL.

^a^Overall (*n* = 7774); Cuban (*n* = 1173), Dominican (*n* = 682), Mexican (*n* = 3354), Puerto Rican (*n* = 1068), Central American (*n* = 876), South American (*n* = 621).

^b^Model 1 adjusts for elapsed time between visits (years) and relevant baseline covariates, including age (years), sex (male, female), self-reported highest education achieved (< high school, high school or equivalent, >high school), nativity (US-born (US states only), foreign-born), total energy intake (kJ/day), and baseline self-reported physical activity level (low, moderate/vigorous) based on days and total metabolic equivalents (minutes/day) assessed using a modified Global Physical Activity Questionnaire, which asks about activity and inactivity (i.e. sedentary behavior) in several life domains (work, travel, leisure).

^c^Model 2 additionally adjusts for baseline waist circumference (cm). *P*_trend_ values were calculated by including the midpoint of each quintile of each heritage-specific *a posteriori* dietary pattern as a continuous variable (score units).

Asterisk (*) indicates *P* < 0.05.

## Discussion

In a large prospective cohort of US Hispanics/Latinos of diverse origin and without diabetes, we identified 5 overarching DPs characterized by foods shared by two or more heritage groups. Depending on the heritage group, some DPs were associated with IR status and diabetes risk 6 years later. For instance, a “Burgers, Fries, & Soft Drinks” DP only in the Dominican group, and a “White Rice, Beans, & Red Meats” DP only in the Cuban group were associated with worsened IR status. While the Puerto Rican “Burger, Fries, & Soft Drinks” DP and the Central American “White Rice, Beans, & Red Meats” DP were associated with increased diabetes risk, only the Puerto Rican “Burger, Fries, & Soft Drinks” DP predicted diabetes, independent of central adiposity.

As previously reported by others [11, 30], DPs characterized by higher intakes of refined grains, processed meats, fried potatoes, and sweet/sugary drinks were associated with increased diabetes risk, whereas DPs higher in fruit, vegetables, poultry, fish, and whole grains were associated with decreased risk. Findings related to IR are similar [7, 31–33]. Most of the foods identified in previous studies were components of the “Burger, Fries, & Soft Drinks”, “White Rice, Beans, & Red Meats”, and “Fish & Whole Grain” DPs in our study. However, these DPs were significantly associated with diabetes and IR status only in some heritage groups. Nonetheless, the direction of the associations among these DPs, including the “Fish & Whole Grain” DPs, tracked in the expected direction for most heritage groups, even though estimates were not statistically significant.

Despite similarly high loadings for the dietary components comprising the five overarching DPs across heritage groups, we suggest a few reasons underlying the statistical significance found for some DPs in some heritage groups, but not others. First, compared to the “Burger, Fries, & Soft Drinks” DPs in other heritage groups, in the Puerto Rican group we found higher negative loadings (lower intakes) of fruit, vegetables, fish, and whole grains, food groups that are generally associated with reduced diabetes risk [11]. Thus, lower intakes of nutrient dense foods and greater intakes of nutrient-poor and energy-dense foods may contribute significantly to higher risk of diabetes associated with this DP, specifically observed in the Puerto Rican group.

Secondly, a caveat of forming food groups based on cultural and behavioral perspectives (as opposed to nutritional or biological value) is that derived DPs may not maximally explain variation in the outcome(s) of interest [34]. For example, the “White Rice, Beans, & Red Meats” DP had high loadings for white rice and beans, each of which have been shown to have contrasting impacts on diabetes and related markers [35, 36]. Similarly, high intakes of these foods in the overall diet may, on average, nullify their overall effect on health. We see evidence of this in our study, as observed by the differences in loadings between white rice and beans, which were notably greater for the Central American and Cuban groups. These higher rice-to-bean ratios may explain why this DP was associated with greater diabetes risk in the Central American group, and worsened IR status among the Cuban group. A previous study among Central Americans, specifically Costa Ricans, found that substituting 1 serving of beans for 1 serving of white rice was associated with decreased risk of the metabolic syndrome [37]. Although the rice and bean loadings were similar in magnitude to each other for the Dominican “White Rice, Beans, & Red Meats” DP, which was also associated with worsened IR status, high loadings were also observed for red meats and starchy vegetables, food groups prominently comprising DPs previously linked to diabetes [38]. These findings suggest that the ratio of intakes of these foods might be a major driver of IR and diabetes risk among Hispanics/Latinos with this type of diet. Future work, however, is needed to verify our hypotheses surrounding rice-and-beans (and other) dietary interactions with respect to IR and diabetes. Additionally, research is warranted to better understand how the rest of the diet plays a role in influencing IR and diabetes risk among Hispanics/Latinos with characteristically similar DPs.

Further, analyses of the DPs with AHEI-2010, may provide additional context for the associations we observed in relation to IR and diabetes risk [30]. In some instances, the same overarching DPs were associated with different levels of healthfulness (measured using AHEI-2010) depending on the heritage group. For example, the “Burger, Fries, & Soft Drinks” DP in Puerto Ricans had among the lowest diet quality scores other heritage groups (Supplementary Fig. 3), potentially explaining the significantly higher risk of diabetes in this group.

We also found that the “Burger, Fries, & Soft Drinks” DP remained associated with diabetes risk among the Puerto Rican heritage group, even after accounting for WC. Also, this suggests that other mechanisms could contribute to the development of diabetes, independent of adiposity. For example, in the Boston Puerto Rican Health Study [39], Mattei and colleagues showed that a DP similar to our “Burger, Fries, & Soft Drinks” DP was associated with higher allostatic load, a risk factor for diabetes, independent of BMI. These findings suggest as health risk associated with a “Burger, Fries, & Soft Drinks” DP beyond its impact on adiposity, at least in those of Puerto Rican heritage. By contrast, the Cuban “Cheese & Sweets” dietary pattern was significantly associated with reduced diabetes risk only after adjusting for WC, suggesting a similar health risk beyond central adiposity for individuals of Cuban heritage. Future work, however, is needed to verify our findings.

Our study is the first to prospectively examine associations among culturally-relevant *a posteriori* DPs, IR, and diabetes risk in different Hispanic/Latino heritage groups. Another strength is the focus on culturally-relevant foods in the derivation of heritage-specific DPs, which better reflect eating patterns among this heterogenous US population. Additionally, HCHS/SOL provided ample sample size for deriving heritage-specific *a posteriori* DPs from two 24 h recalls and examining relationships with IR and diabetes.

This study has some limitations. First, because DPs were derived separately by heritage, we were unable to formally evaluate heterogeneity of associations between DPs and outcomes by heritage. However, as a first step in this line of research, this study explored the role of dietary culturally-relevant dietary patterns on IR and diabetes risk and demonstrated associations for some heritage groups but not others. For instance, the combinations of foods in the “Burgers, Fries, & Soft Drinks” DP among individuals of Puerto Rican heritage may be particularly important for the development of diabetes, but in this group, replication of these findings is first needed before formulating heritage-specific dietary guidance. Second, since DPs were derived focusing on cultural and behavioral over nutrient value, ingredients/foods with contrasting effects on diabetes outcomes (e.g., white rice and beans) may have biased estimates towards the null. Future studies should investigate which combinations of dietary components (e.g., foods, nutrients) of similar DPs are important dietary drivers of IR and diabetes. Future studies should also evaluate the extent to which the source/origin of foods (e.g., commercial vs. homemade burgers) in, for example, the overarching “Burgers, Fries, & Soft Drinks” and other DPs contribute to IR status and diabetes risk. Third, we could not account for changes in diet over 6 years, which could influence IR status and diabetes risk. Fourth, we also cannot rule out the role of selection bias. In our sensitivity analyses, compared to the analytic sample, excluded participants were younger, male, US-born, less likely to report an education beyond HS, and had lower mean AHEI-2010 diet quality scores. Several of these factors have been shown to increase diabetes risk. Although it is difficult to determine the direction of the bias, because excluded participants had risk factors that generally placed them at greater risk of developing diabetes, our findings may represent underestimates of true associations. Lastly, we did not distinguish between type 1 and type 2 diabetes. However, because only 8.3% of incident diabetes occurred among those aged 18–34 years (author’s calculations), it is likely that the majority of subjects with incident diabetes developed type 2 diabetes.

## Conclusions

Taken together, these findings suggest that a diet characterized by higher intakes of burgers, fries, and soft drinks and another characterized by higher intakes of white rice, beans, and red meats may be adversely associated with IR and diabetes risk in certain Hispanic/Latino heritage groups. Future work evaluating combinations of foods that may be important drivers of IR and diabetes risk in these and other similar diets are needed to tailor dietary interventions by Hispanic/Latino heritage for diabetes prevention. Nevertheless, our findings can inform future research on identifying optimal intakes of different food combinations for application in dietary clinical trials. Given the fast growth of this population [40], coupled with the disproportionate risk of diabetes faced by Hispanics/Latinos, identifying effective strategies tailored to different Hispanic/Latino heritage groups will be crucial.

## Supplementary information

Supplemental Tables S1-S3

Supplemental Figure 1

Supplemental Figure 2

Supplemental Figure 3

## Data Availability

The data and computer code used for this analysis reside at UNC Chapel Hill. The HCHS/SOL fully supports data sharing with outside investigators through processes internal to the study, based on a Data and Materials Distribution Agreement (DMDA) to protect the confidentiality and privacy of the HCHS/SOL participants and their families. Alternatively, de-identified HCHS/SOL data are publicly available at BioLINCC and dbGaP for the subset of the study cohort that authorized general use of their data at the time of informed consent. Data described in the manuscript, code book, and analytic code will be made available upon reasonable request pending IRB review and approval.

## Code availability

Data described in the manuscript, code book, and analytic code will be made available upon reasonable request pending IRB review and approval.
