# Supplementary material for: A Posteriori dietary patterns, insulin resistance, and diabetes risk by Hispanic/Latino heritage in the HCHS/SOL cohort
Source: Nutr Diabetes. 2022 Oct 13;12:44. doi: 10.1038/s41387-022-00221-3 (PMC9561638; doi:10.1038/s41387-022-00221-3)
Supplement: Supplementary file 1 — Supplemental Tables S1-S3 [file 41387_2022_221_MOESM1_ESM.docx]

**Supplemental Table S1.** Food groups used in heritage-specific principal factor analyses among HCHS/SOL participants with two reliable/plausible 24-hour dietary recalls, non-missing data on relevant heritage groups, and without diabetes at baseline (n=11,125)

| Food Groups (n=34) | Examples^a^ | Median (IQRs)^b^ | NC (%)^c^ |
| --- | --- | --- | --- |
| Cheese | queso fresco, farmer cheese, American cheese | 308.8 (169.5) | 88.7 |
| Milk | unflavored milk^c^, yogurt, flavored milk | 354 (150.5) | 51.1 |
| Fruit | bananas, apples, avocado, grapes | 98.7 (109.6) | 47.1 |
| Non-starchy vegetables | tomato, mixed veg, lettuce, broccoli | 58.6 (107.9) | 71.1 |
| Starchy vegetables | plantains, yuca blanca, mashed potatoes, potatoes | 91.5 (201.3) | 68.5 |
| Salads | tossed salads, lettuce salads, Caesar salads | 21.3 (28.3) | 69.3 |
| Fish | shrimp, tilapia, salmon, bacalao | 74.3 (213.9) | 85.0 |
| Poultry | chicken (breast, drumstick, leg, wing) | 49.1 (72) | 58.7 |
| Pork | pork (chops, steak, ribs, cubes) | 88 (75.2) | 86.2 |
| Beef | bistec (steak casserole), beef (steak, sirloin, meatballs) | 85 (117.3) | 72.5 |
| Processed meats | bacon, sausage, ham, salami, corned beef | 50.3 (67.5) | 85.8 |
| Burgers | hamburger or ground beef and buns, big mac | 99 (109) | 90.3 |
| Fries | french fries^c^, hashed brown potatoes | 118 (106) | 85.0 |
| Fried dishes | fried foods (e.g., fried chicken), empanadas | 60 (67.5) | 82.3 |
| Pizza | pizza | 112 (115.3) | 87.6 |
| Dessert | pan dulce, ice cream, cookies, cake | 170.1 (253.5) | 44.4 |
| Sweets | ice popsicle, chocolate candy (e.g. snickers, bar, kit kat) | 103 (292.6) | 83.3 |
| Salty snacks | crackers, tortilla/nacho chips, potato chips | 149 (219) | 66.1 |
| Refined grains | white bread, bagels, flour tortillas | 212.6 (303.5) | 54.7 |
| Cereal | ready-to-eat cereals & milk^c^, with or without milk | 151.7 (97) | 65.0 |
| Whole grains | whole wheat bread^c^ | 85 (83.9) | 82.9 |
| Corn-based foods | corn tortillas^c^, tacos, tamales | 86 (62) | 56.3 |
| Meat & vegetable stews | carne guisada (beef), picadillo de carne (meat & tomato) | 41 (59.6) | 76.7 |
| Beans | beans (pinto, black, red)^c^, lentils | 36 (57.4) | 58.5 |
| Eggs | scrambled eggs, fried eggs, boiled eggs | 158 (158) | 68.1 |
| Rice | white rice^c^, rice and beans | 67.5 (90.3) | 33.7 |
| Noodle-based | spaghetti noodles & sauce, ramen, macaroni & cheese | 68 (93) | 77.3 |
| Sandwiches & rolls | sandwiches^c^, hot dogs, burritos | 19.6 (20.5) | 51.5 |
| Soups | chicken & veg soup, noodle/pasta soup, pozole | 41.6 (68.3) | 69.7 |
| Soft beverages | (Coke, Pepsi, Sprite)^c^, diet soda | 355.2 (138.6) | 45.5 |
| Flavored drinks | fruit juices or drinks (e.g. orange, apple)^c^, Gatorade | 310.7 (419.4) | 34.6 |
| Coffee/tea | coffee & milk/cream^c^, tea (with or without milk/cream) | 114 (153) | 28.4 |
| Alcoholic beverages | beer^c^, wine, spirits | 42 (81.9) | 84.1 |
| Water | bottled water^c^, mineral water, tap water | 500.7 (234) | 08.1 |
| Miscellaneous^d^ | herbalife shakes^c^, sour cream, energy drinks | 28.4 (42.5) | 90.4 |

Hispanic Community Health Survey/Study of Latinos, HCHS/SOL; Interquartile range, IQR; Non-consumers, NCs

Food groups are total intakes (g) averaged across two, non-consecutive 24-hour recalls in HCHS/SOL

^a^ Examples are listed in descending order of frequency in their food group

^b^ Median intakes (g) in the overall sample defined 3-level ordinal intake variables (NCs, below & above median intakes)

^c^ Most represented food in their food group

^d^ Dropped from analysis due to consumption < 5% found in at least one Hispanic/Latino heritage group

**Supplemental Table S2.** Baseline sociodemographic characteristics and AHEI-2010 scores by inclusion/exclusion criteria among Hispanics/Latinos without diabetes at baseline (n=12,995) in the HCHS/SOL

| Sociodemographics | Analytic | Excluded^a^ | *P* |
| --- | --- | --- | --- |
|  | n=7,774 | n=5,221 |  |
| Age (years) | 41.3 ± 0.3 | 35.7 ± 0.3 | <0.001 |
| Female (%) | 56.0 | 46.7 | <0.001 |
| US-Born (%) | 19.3 | 31.3 | <0.001 |
| Education (%) |  |  | <0.001^b^ |
| < High school | 29.3 | 30.5 | 0.295 |
| High school/equivalent | 28.3 | 30.3 | 0.100 |
| > High school | 42.3 | 39.2 | 0.013 |
| Heritage group (%) |  |  | <0.001^b^ |
| Cuban | 9.5 | 11.6 | 0.024 |
| Dominican | 7.8 | 7.9 | 0.864 |
| Mexican | 21.1 | 20.1 | 0.399 |
| Puerto Rico | 42.2 | 35.6 | <0.001 |
| Central American | 13.4 | 19.7 | <0.001 |
| South American | 5.9 | 5.1 | 0.092 |
| AHEI-2010 (scores)^c^ | 47.9 ± 0.2 | 45.7 ± 0.2 | <0.001 |

Values are means ± SEs unless otherwise noted. All analyses were survey-weighted. Sample sizes are unweighted.

^a^ Excluded participants were those who fasted < 8-hrs prior to either clinic visit, had at least one recall with estimated energy intakes below or above their corresponding 1^st^ and 99^th^ sex-specific percentiles in HCHS/SOL or were deemed unreliable by an interviewer, self-reported heritage as more than one or other, or had missing data on these or any other relevant covariates.

^b^  Overall *P*-value

^c^ Alternative Healthy Eating Index (2010)

**Supplemental Table S3.** *A posteriori* heritage-specific principal factor loadings by overarching dietary patterns identified across Hispanic/Latino heritage groups without diabetes at baseline in HCHS/SOL (n=11,125)

| Food Groups (n=34) | “Burger, Fries, & Soft Drinks” | | | | | | “White Rice, Beans, & Red Meats” | | | | |
| --- | --- | --- | --- | --- | --- | --- | --- | --- | --- | --- | --- |
|  | CB | DM | MX | PR | CA | SA | CB | DM | MX | PR | CA |
|  | F1 | F1 | F1 | F1 | F1 | F2 | F3 | F2 | F3 | F2 | F3 |
| Cheese | -0.17 | -0.20 | -0.16 | -0.13 | -0.05 | 0.05 | 0.21 | 0.24 | 0.21 | 0.06 | 0.05 |
| Milk | -0.08 | -0.10 | -0.11 | -0.14 | -0.14 | 0.14 | -0.07 | -0.03 | -0.09 | 0.15 | 0.06 |
| Fruit | -0.10 | -0.30 | -0.26 | -0.30 | -0.32 | -0.12 | -0.14 | -0.23 | -0.06 | 0.03 | -0.21 |
| Non-starchy Vegetables | -0.15 | -0.28 | -0.19 | -0.26 | -0.21 | -0.11 | -0.06 | -0.19 | 0.24 | 0.02 | 0.01 |
| Starchy Vegetables | -0.23 | -0.25 | -0.05 | -0.16 | -0.13 | -0.21 | -0.01 | 0.53 | 0.23 | 0.02 | 0.30 |
| Salads | -0.15 | -0.21 | -0.09 | -0.24 | -0.23 | -0.19 | -0.05 | -0.09 | 0.02 | 0.05 | 0.11 |
| Fish | -0.13 | -0.10 | -0.06 | -0.27 | -0.28 | -0.10 | 0.00 | -0.23 | 0.16 | -0.20 | 0.18 |
| Poultry | -0.02 | 0.07 | -0.05 | -0.10 | -0.08 | -0.17 | -0.18 | 0.17 | 0.21 | 0.18 | 0.22 |
| Pork | -0.20 | 0.06 | -0.04 | 0.10 | 0.10 | -0.21 | 0.42 | 0.35 | 0.22 | 0.49 | 0.09 |
| Beef | -0.04 | 0.05 | -0.03 | 0.05 | 0.03 | -0.18 | -0.06 | 0.36 | 0.28 | 0.22 | 0.57 |
| Processed Meats | -0.05 | -0.05 | 0.19 | 0.12 | 0.13 | 0.04 | 0.32 | 0.39 | 0.29 | 0.09 | -0.01 |
| Burgers | 0.80 | 0.65 | 0.69 | 0.70 | 0.75 | 0.78 | -0.06 | -0.19 | -0.08 | -0.02 | -0.13 |
| Fries | 0.56 | 0.66 | 0.71 | 0.66 | 0.66 | 0.60 | 0.08 | -0.09 | 0.02 | -0.07 | -0.07 |
| Fried | 0.16 | 0.46 | 0.20 | 0.18 | 0.08 | -0.02 | 0.14 | -0.04 | 0.07 | -0.12 | 0.15 |
| Pizza | 0.36 | 0.53 | 0.26 | 0.25 | 0.22 | 0.23 | 0.10 | 0.04 | -0.14 | -0.23 | -0.05 |
| Dessert | 0.07 | 0.18 | 0.03 | -0.05 | 0.18 | -0.05 | 0.05 | 0.07 | 0.07 | 0.09 | -0.04 |
| Sweets | -0.02 | 0.13 | 0.06 | 0.03 | 0.02 | 0.03 | -0.09 | 0.07 | 0.04 | 0.00 | -0.16 |
| Salty Snacks | -0.08 | -0.07 | 0.22 | 0.09 | 0.01 | 0.17 | 0.12 | -0.12 | 0.06 | -0.08 | -0.11 |
| Refined Grains | 0.14 | 0.08 | 0.09 | 0.07 | 0.11 | -0.12 | 0.10 | -0.02 | 0.19 | 0.05 | 0.05 |
| Cereal | -0.07 | -0.12 | -0.08 | -0.18 | -0.22 | 0.04 | -0.27 | -0.16 | -0.22 | -0.01 | -0.12 |
| Whole Grains | -0.03 | -0.23 | -0.13 | -0.24 | -0.36 | -0.05 | -0.33 | -0.18 | -0.06 | -0.10 | -0.24 |
| Corn-based | 0.02 | 0.10 | -0.20 | 0.14 | 0.12 | 0.06 | 0.08 | -0.19 | 0.04 | -0.11 | -0.12 |
| Meat & Vegetable Stew | -0.25 | -0.10 | -0.17 | 0.07 | -0.19 | -0.08 | 0.00 | -0.12 | 0.08 | 0.18 | 0.18 |
| Beans | -0.11 | -0.10 | -0.19 | -0.09 | -0.08 | -0.21 | 0.10 | 0.31 | 0.39 | 0.55 | 0.20 |
| Eggs | -0.04 | -0.07 | 0.02 | 0.16 | 0.09 | 0.00 | 0.07 | 0.49 | 0.27 | 0.13 | 0.06 |
| White Rice | -0.22 | -0.14 | -0.15 | -0.05 | -0.09 | -0.24 | 0.22 | 0.31 | 0.31 | 0.83 | 0.54 |
| Noodle-based | 0.27 | 0.06 | 0.14 | 0.00 | 0.10 | -0.09 | -0.09 | -0.13 | -0.07 | -0.11 | -0.05 |
| Sandwiches | 0.35 | 0.43 | 0.29 | 0.21 | 0.19 | 0.35 | 0.19 | 0.00 | -0.12 | -0.13 | -0.20 |
| Soups | -0.10 | -0.06 | -0.16 | -0.29 | -0.04 | -0.21 | -0.12 | -0.21 | -0.20 | -0.12 | -0.37 |
| Soft drinks | 0.52 | 0.76 | 0.42 | 0.58 | 0.63 | 0.31 | 0.52 | 0.05 | 0.17 | -0.05 | 0.38 |
| Flavored drinks | -0.04 | 0.14 | 0.02 | 0.02 | 0.04 | 0.01 | 0.09 | 0.23 | 0.00 | 0.08 | 0.04 |
| Coffee/Tea | -0.05 | 0.01 | -0.06 | -0.22 | -0.05 | -0.12 | -0.13 | -0.25 | 0.16 | 0.00 | -0.13 |
| Alcohol | 0.00 | 0.24 | 0.21 | 0.24 | 0.22 | 0.03 | 0.40 | 0.18 | 0.27 | -0.07 | 0.28 |
| Water | -0.07 | -0.30 | -0.15 | -0.13 | -0.27 | -0.07 | -0.13 | -0.01 | -0.05 | 0.03 | -0.07 |
| *Variance (%)*^b^ | 15.9 | 15.8 | 26.6 | 16.5 | 12.9 | 13.5 | 8.1 | 9.8 | 15.0 | 11.4 | 7.9 |

*(CONTINUED)*

**Supplemental Table S3.** *(CONTINUED)*

| Food Groups (n=34) | “Fish & Whole Grain” | | | | “Cheese & Sweets” | | “Stew & Corn” | |
| --- | --- | --- | --- | --- | --- | --- | --- | --- |
|  | CB | MX | PR | SA | CB | SA | PR | CA |
|  | F4 | F2 | F3 | F1 | F2 | F3 | F4 | F2 |
| Cheese | 0.26 | 0.02 | 0.31 | 0.15 | 0.43 | 0.29 | 0.18 | 0.45 |
| Milk | -0.09 | 0.02 | 0.01 | 0.17 | -0.02 | -0.10 | 0.12 | 0.03 |
| Fruit | 0.12 | 0.22 | 0.19 | 0.28 | 0.19 | -0.03 | 0.21 | -0.05 |
| Non-starchy Vegetables | 0.10 | 0.21 | 0.24 | 0.33 | -0.07 | 0.19 | -0.01 | -0.14 |
| Starchy Vegetables | 0.17 | 0.21 | 0.28 | 0.02 | -0.10 | 0.18 | -0.17 | -0.05 |
| Salads | 0.22 | 0.46 | 0.11 | 0.13 | 0.01 | 0.04 | 0.03 | -0.16 |
| Fish | 0.50 | 0.27 | 0.41 | 0.30 | 0.00 | 0.12 | 0.03 | -0.31 |
| Poultry | 0.47 | 0.31 | 0.18 | -0.09 | -0.05 | 0.22 | -0.62 | -0.17 |
| Pork | -0.13 | -0.10 | -0.21 | -0.45 | -0.07 | -0.15 | -0.07 | 0.11 |
| Beef | 0.10 | -0.04 | -0.07 | -0.06 | -0.49 | 0.13 | -0.08 | 0.01 |
| Processed Meats | 0.21 | 0.06 | 0.46 | -0.08 | 0.17 | 0.15 | -0.17 | 0.13 |
| Burgers | 0.01 | 0.02 | 0.00 | -0.02 | 0.10 | -0.02 | 0.20 | -0.04 |
| Fries | 0.05 | 0.02 | 0.09 | -0.13 | -0.12 | 0.04 | 0.04 | -0.11 |
| Fried | -0.03 | -0.01 | 0.11 | -0.22 | 0.20 | 0.23 | -0.11 | -0.09 |
| Pizza | -0.02 | -0.02 | -0.16 | -0.10 | 0.13 | -0.07 | -0.05 | -0.51 |
| Dessert | -0.02 | -0.08 | -0.03 | -0.04 | 0.12 | 0.24 | 0.04 | 0.05 |
| Sweets | 0.10 | 0.15 | 0.15 | 0.13 | 0.23 | 0.33 | 0.12 | -0.12 |
| Salty Snacks | 0.05 | 0.13 | 0.18 | 0.22 | 0.12 | 0.08 | 0.12 | -0.07 |
| Refined Grains | -0.07 | 0.09 | 0.03 | -0.21 | -0.05 | 0.21 | -0.02 | 0.08 |
| Cereal | 0.12 | 0.22 | -0.11 | 0.35 | 0.11 | -0.09 | 0.14 | -0.18 |
| Whole Grains | 0.20 | 0.30 | 0.26 | 0.54 | 0.11 | 0.07 | 0.09 | -0.08 |
| Corn-based | 0.00 | -0.49 | 0.13 | -0.10 | 0.28 | -0.02 | 0.27 | 0.45 |
| Meat & Vegetable Stew | -0.55 | -0.26 | 0.11 | -0.09 | -0.09 | -0.39 | 0.63 | 0.33 |
| Beans | 0.07 | -0.23 | -0.02 | -0.06 | -0.40 | -0.14 | -0.09 | 0.42 |
| Eggs | -0.05 | -0.10 | 0.36 | -0.12 | 0.00 | 0.22 | -0.12 | 0.37 |
| White Rice | -0.09 | 0.10 | 0.04 | -0.29 | -0.53 | -0.29 | 0.04 | 0.14 |
| Noodle-based | -0.17 | 0.17 | -0.14 | -0.04 | 0.23 | 0.30 | 0.01 | -0.25 |
| Sandwiches | 0.14 | -0.01 | -0.22 | -0.08 | 0.07 | 0.21 | -0.06 | -0.26 |
| Soups | -0.21 | -0.07 | -0.02 | 0.00 | 0.06 | -0.27 | 0.16 | 0.25 |
| Soft drinks | -0.03 | -0.35 | -0.15 | -0.58 | -0.10 | 0.11 | -0.13 | 0.07 |
| Flavored drinks | 0.11 | 0.01 | -0.06 | -0.08 | 0.00 | 0.00 | 0.02 | -0.05 |
| Coffee/Tea | 0.04 | 0.09 | 0.07 | -0.05 | 0.19 | 0.22 | 0.08 | 0.09 |
| Alcohol | 0.02 | -0.03 | 0.33 | -0.08 | 0.10 | 0.43 | 0.02 | -0.07 |
| Water | 0.11 | 0.15 | 0.24 | 0.39 | -0.08 | -0.08 | 0.19 | 0.12 |
| *Variance (%)*^b^ | 7.9 | 17.6 | 8.8 | 16.1 | 11.7 | 11.7 | 10.0 | 8.3 |

Hispanic Community Health Survey/Study of Latino, HCHS/SOL;

^a^ Cuban (CB, n=1,722), Dominican (DM, n=1,019), Mexican (MX, n=4,592), Puerto Rican (PR, n=1,677), Central American (CA, n=1,262), South American (SA, n=853); Factor, F. All factors were rotated orthogonally (varimax) and factor loadings ≥ 0.20 only show for simplicity and those ≥ 0.30 are bolded. Data are from heritage-specific principal factor analyses performed on polychoric correlation matrixes of ordinal food groups (nonconsumers, and below and above the median intake (grams) in the overall sample). Food groups (grams/day) were formed and averaged using two, nonconsecutive 24-hour dietary recalls from each participant

^b^ Variance explained by each derived factor

**FIGURES**

**Supplemental Figure S1.** Flow chart of participants without diabetes at baseline for heritage-specific principal factor analyses in HCHS/SOL. **Footnote.** Data are from the Hispanic/Latino Community Health Survey/Study of Latinos (HCHS/SOL). ^a^ Estimated energy intake was below or above the sex-corresponding 1^st^ and 99^th^ percentiles in HCHS/SOL or deemed unreliable by the interviewer

**Supplemental Figure S2.** Heritage-specific scree plots from principal factor analyses by Hispanic/Latino heritage without diabetes at baseline. **Footnote.** Scree plots display derived eigenvalues and factor numbers from each principal factor analysis in each Hispanic/Latino heritage group: Cuban (n=1,722), Dominican (n=1,019), Mexican (n=4,592), Puerto Rican (n=1,677), Central American (n=1,262), South American (n=853). Horizontal line symbolizes Kaiser criterion for factor retention (eigenvalues > 1). Principal factor analyses were performed on heritage-specific matrixes of polychoric correlations between 34 ordinal food group intake variables (nonconsumers, below and above the median) using food group intakes (grams/day) in the overall sample (n=11,125).

**Supplemental Figure S3.** Mean AHEI-2010 scores by quintiles of *a posteriori* heritage-specific dietary patterns for each overarching dietary pattern identified among Hispanics/Latinos in HCHS/SOL without diabetes at baseline. **Footnote**. Data presented are weighted mean AHEI-2010 score differences (95% confidence intervals) by quintiles (Qs) of each heritage-specific dietary pattern from multivariable linear regression models showing mean AHEI-2010 score differences comparing higher-to-lowest quintiles (Q2-Q5 vs. Q1), adjusting for age (years), sex (male, female), highest education achieved (less than high school, high school or equivalent, beyond high school), and other heritage-specific dietary patterns (quintiles). Asterisks (∗) indicate statistically significant pairwise comparisons of higher-versus-lowest quintiles of each heritage-specific dietary pattern (*P*<0.05). Asterisks (∗) on heritage group name indicate statistically significant linear trends (all *P*_trend_<0.001), which were tested by using the midpoint of each dietary pattern quintile as a continuous measure. Cuban (CB, n=1,173), Dominican (DM, n=682), Mexican (MX, n=3,354), Puerto Rican (PR, n=1,068), Central American (CA, n=876), South American (SA, n=621).
