# Supplementary material for: A Posteriori dietary patterns, insulin resistance, and diabetes risk by Hispanic/Latino heritage in the HCHS/SOL cohort
Source: Nutr Diabetes. 2022 Oct 13;12:44. doi: 10.1038/s41387-022-00221-3 (PMC9561638; doi:10.1038/s41387-022-00221-3)
Supplement: Supplementary file 2 — Supplemental Figure 1 [file 41387_2022_221_MOESM2_ESM.pdf]

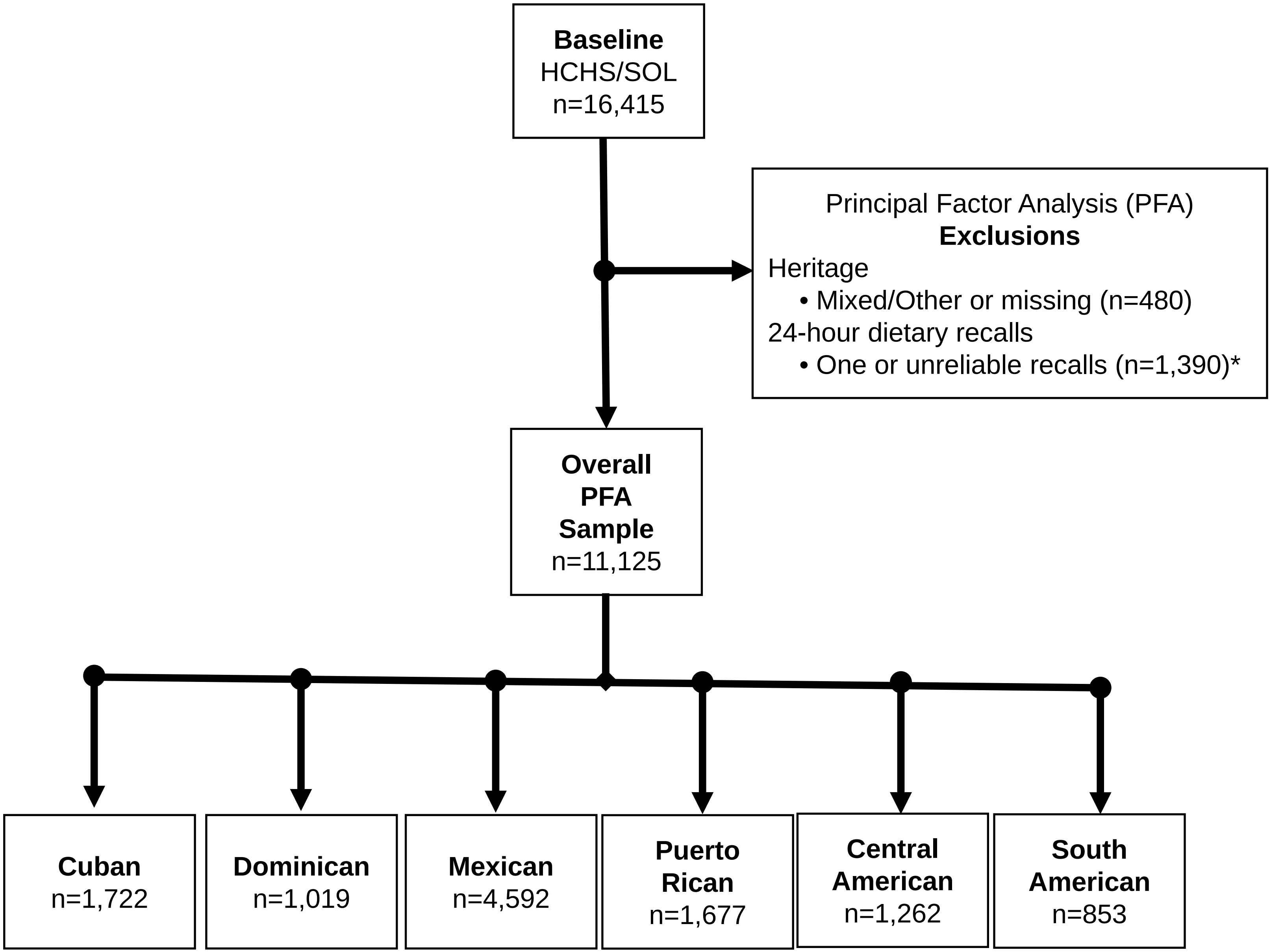

Baseline
HCHS/SOL
n=16,415
Principal Factor Analysis (PFA)
Exclusions
Heritage
• Mixed/Other or missing (n=480)
24-hour dietary recalls
• One or unreliable recalls (n=1,390)*
Overall PFA Sample
n=11,125
Cuban
n=1,722
Dominican
n=1,019
Mexican
n=4,592
Puerto Rican
n=1,677
Central American
n=1,262
South American
n=853
