# Supplementary figures and images for: A Posteriori dietary patterns, insulin resistance, and diabetes risk by Hispanic/Latino heritage in the HCHS/SOL cohort
Source: Nutr Diabetes. 2022 Oct 13;12:44. doi: 10.1038/s41387-022-00221-3 (PMC9561638; doi:10.1038/s41387-022-00221-3)

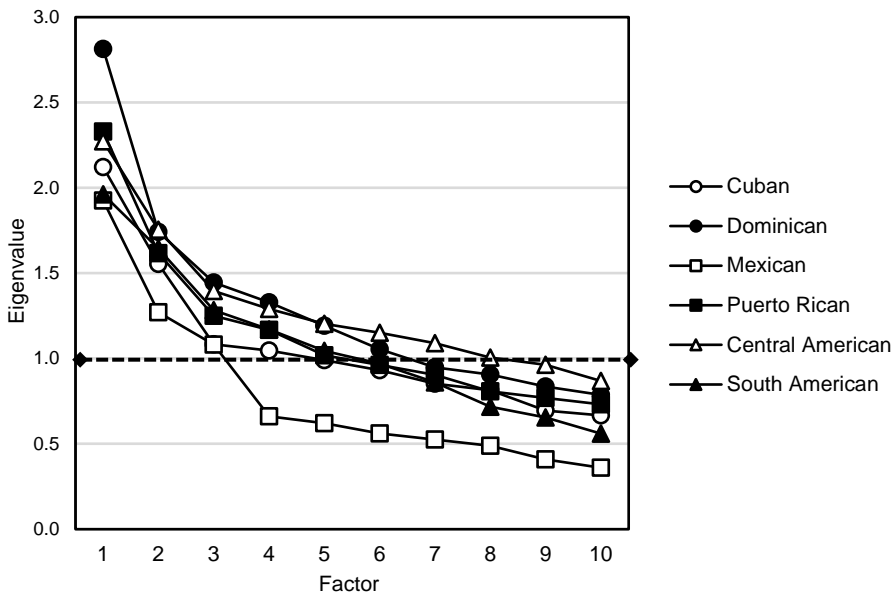

Supplement: Supplementary file 3 — Supplemental Figure 2 [file 41387_2022_221_MOESM3_ESM.pdf]

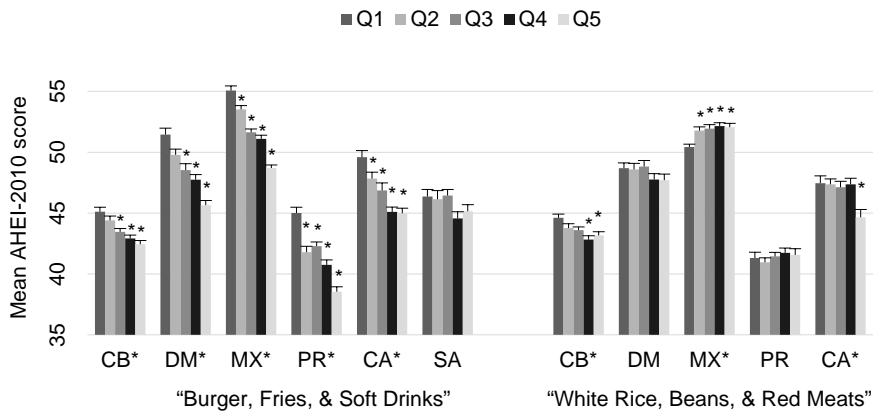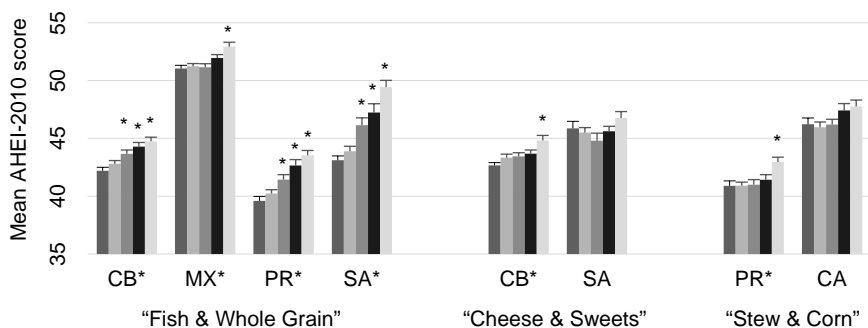

Supplement: Supplementary file 4 — Supplemental Figure 3 [file 41387_2022_221_MOESM4_ESM.pdf]
